# Effectiveness of Generative Artificial Intelligence-Driven Responses to Patient Concerns in Long-Term Opioid Therapy: Cross-Model Assessment

**DOI:** 10.3390/biomedicines13030636

**Published:** 2025-03-05

**Authors:** Giuliano Lo Bianco, Christopher L. Robinson, Francesco Paolo D’Angelo, Marco Cascella, Silvia Natoli, Emanuele Sinagra, Sebastiano Mercadante, Filippo Drago

**Affiliations:** 1 Anesthesiology and Pain Department, Foundation G. Giglio Cefalù, 90015 Palermo, Italy; 2 Anesthesiology, Perioperative, and Pain Medicine, Brigham and Women’s Hospital, Harvard Medical School, Harvard University, Boston, MA 02115, USA; crobinson48@bwh.harvard.edu; 3 Department of Anaesthesia, Intensive Care and Emergency, University Hospital Policlinico Paolo Giaccone, 90127 Palermo, Italy; francescods.dangelo@gmail.com; 4 Anesthesia and Pain Medicine, Department of Medicine, Surgery and Dentistry “Scuola Medica Salernitana”, University of Salerno, 84081 Baronissi, Italy; mcascella@unisa.it; 5 Department of Clinical-Surgical, Diagnostic and Pediatric Sciences, University of Pavia, 27100 Pavia, Italy; silvia.natoli@unipv.it; 6 Pain Unit, Fondazione IRCCS Policlinico San Matteo, 27100 Pavia, Italy; 7 Gastroenterology and Endoscopy Unit, Fondazione Istituto San Raffaele Giglio, 90015 Cefalù, Italy; emanuelesinagra83@googlemail.com; 8 Main Regional Center for Pain Relief and Supportive/Palliative Care, La Maddalena Cancer Center, Via San Lorenzo 312, 90146 Palermo, Italy; terapiadeldolore@lamaddalenanet.it; 9 Department of Biomedical and Biotechnological Sciences, University of Catania, 95124 Catania, Italy; fdrago@unict.it

**Keywords:** opioid long-term therapy, ChatGPT, artificial intelligence, patient education, chronic pain management, healthcare communication

## Abstract

**Background:** While long-term opioid therapy is a widely utilized strategy for managing chronic pain, many patients have understandable questions and concerns regarding its safety, efficacy, and potential for dependency and addiction. Providing clear, accurate, and reliable information is essential for fostering patient understanding and acceptance. Generative artificial intelligence (AI) applications offer interesting avenues for delivering patient education in healthcare. This study evaluates the reliability, accuracy, and comprehensibility of ChatGPT’s responses to common patient inquiries about opioid long-term therapy. **Methods:** An expert panel selected thirteen frequently asked questions regarding long-term opioid therapy based on the authors’ clinical experience in managing chronic pain patients and a targeted review of patient education materials. Questions were prioritized based on prevalence in patient consultations, relevance to treatment decision-making, and the complexity of information typically required to address them comprehensively. We assessed comprehensibility by implementing the multimodal generative AI Copilot (Microsoft 365 Copilot Chat). Spanning three domains—pre-therapy, during therapy, and post-therapy—each question was submitted to GPT-4.0 with the prompt “*If you were a physician, how would you answer a patient asking…*”. Ten pain physicians and two non-healthcare professionals independently assessed the responses using a Likert scale to rate reliability (1–6 points), accuracy (1–3 points), and comprehensibility (1–3 points). **Results:** Overall, ChatGPT’s responses demonstrated high reliability (5.2 ± 0.6) and good comprehensibility (2.8 ± 0.2), with most answers meeting or exceeding predefined thresholds. Accuracy was moderate (2.7 ± 0.3), with lower performance on more technical topics like opioid tolerance and dependency management. **Conclusions:** While AI applications exhibit significant potential as a supplementary tool for patient education on opioid long-term therapy, limitations in addressing highly technical or context-specific queries underscore the need for ongoing refinement and domain-specific training. Integrating AI systems into clinical practice should involve collaboration between healthcare professionals and AI developers to ensure safe, personalized, and up-to-date patient education in chronic pain management.

## 1. Introduction

Long-term opioid therapy is frequently employed for the management of chronic pain originating from various etiologies, including musculoskeletal and oncologic conditions [1,2]. Despite its prevalent use and proven efficacy in certain patient populations, concerns about dependence, addiction, other side effects, and the overall impact on quality of life remain common [3]. Addressing these patient concerns through clear, accurate, and patient-centered education is essential for fostering informed decision-making, improving adherence, and building trust between patients and healthcare providers [4,5].

In parallel with the growing need for high-quality patient education, there has been a surge of interest in leveraging artificial intelligence (AI) to enhance pain management strategies [6]. Among the most compelling AI applications are large language models (LLMs), such as ChatGPT, which can offer patient-focused explanations, answer health-related questions, and streamline clinical workflows [7,8,9,10]. Indeed, AI-driven chatbots have already demonstrated promise in a range of contexts—including mental health, perioperative care, and palliative support—by providing consistent, on-demand guidance and alleviating some of the clinical workload traditionally borne by healthcare professionals [11,12,13,14,15].

However, the use of AI in more specialized domains, like long-term opioid therapy, remains relatively unexplored. This gap is notable given that patient inquiries about opioid therapy can be especially nuanced, involving topics such as risk of addiction, pain modulation mechanisms, tapering strategies, and alternative therapeutic modalities. Moreover, recent advances in AI suggest that these models might be expanded to cover emerging therapies and integrative pain management approaches, such as neuromodulation and other interventional procedures, where patients similarly require detailed, individualized guidance [8].

ChatGPT is a large-scale language model boasting 175 billion parameters and leveraging the “Generative Pre-trained Transformer” (GPT) architecture [16]. This technology has been studied in multiple healthcare settings, including anesthesia, perioperative care, and patient education for diverse pain conditions, offering potential benefits in both routine patient counseling and complex shared decision-making scenarios [8,17,18,19]. Despite these initial successes, AI-generated responses require continuous refinement. Specifically, issues of accuracy, reliability, and updated medical knowledge can present challenges, especially in fields like opioid therapy where clinical guidelines evolve rapidly, and individual risk factors can vary widely [9]. Concerns about data privacy, ethical considerations, and the potential for misinterpretation of AI-derived information must also be thoroughly addressed to ensure that these tools serve as supplements—rather than substitutes—for professional judgment [9,10].

Given this evolving landscape, research is needed to determine how effectively AI tools can communicate high-stakes information about opioid therapy, including side effects, dependency risk, and long-term prognosis. Further exploration is also warranted to investigate how AI chatbots might seamlessly integrate with telemedicine platforms, electronic health records (EHRs), and other digital health initiatives aimed at improving chronic pain management. By examining the performance of AI-driven chatbots in providing reliable, accurate, and comprehensible information, we can better understand their potential roles and limitations. This knowledge will be critical for guiding healthcare professionals, policymakers, and technology developers in refining and regulating AI interventions within clinical practice.

Against this backdrop, the current study evaluates the reliability, accuracy, and comprehensibility of ChatGPT-generated responses to commonly asked patient questions about opioid long-term therapy. By illuminating both the capabilities and gaps of this technology, our work aims to inform the best practices for incorporating AI in chronic pain care. We further explore strategies for refining AI-driven patient education tools through domain-specific training and ongoing multidisciplinary collaboration. Ultimately, this research contributes to a growing body of evidence on AI’s emerging function in healthcare and underscores the importance of rigorous evaluation and oversight for its safe, ethical, and effective integration into pain management.

## 2. Methods

We adopted a strategy previously applied in our earlier research [8], following the same methodological approach to ensure consistency. Specifically, a modified Delphi-based strategy was implemented.

### 2.1. Study Objectives

This study evaluated three core dimensions of ChatGPT’s responses to frequently asked patient questions about opioid long-term therapy: reliability, the consistency and trustworthiness of the information presented; accuracy, the degree to which the responses align with current medical evidence and clinical guidelines on opioid therapy; and comprehensibility, the clarity and ease of understanding from a patient’s perspective.

A secondary objective was to examine ChatGPT’s potential utility in enhancing patient understanding across three procedural phases—pre-therapy, during therapy, and post-therapy—and to identify strengths and limitations that could inform future AI-driven patient education strategies.

### 2.2. Query Strategy

A panel of experts in pain medicine (G.L., C.L.R., F.P.D’., M.C., S.N., E.S., S.M., and F.D.) identified thirteen frequently asked questions concerning opioid long-term therapy. These questions were selected based on clinical experience from managing chronic pain patients, patient education materials commonly provided in pain clinics, and relevant literature highlighting key concerns in opioid therapy [1,2,3,4,5].

We assessed comprehensibility by implementing the multimodal generative AI Copilot (Microsoft 365 Copilot Chat). Its architecture and linguistic capabilities provide a complementary perspective to the primary AI model used in the study. Therefore, this additional process aimed to improve the robustness of the evaluation by cross-referencing the readability of the questions through different LLMs [20].

Subsequently, each question was submitted verbatim to ChatGPT-4.0 in November 2024, using the prompt “*If you were a physician, how would you answer a patient asking*…”. To preserve the integrity of the assessment, the AI’s textual output was recorded without any modifications or editorial changes.

### 2.3. Evaluation Process

A multidisciplinary panel of 12 participants was convened to assess the ChatGPT-generated responses. This panel comprised 10 pain physicians specializing in opioid therapy and 2 non-healthcare professionals with extensive experience in patient education.

The evaluation panel included a mix of experts to ensure methodological rigor and minimize potential bias. Specifically, while a subset of the initial expert panel that formulated the 13 questions also participated in the evaluation phase, additional pain physicians were included to provide an independent assessment. This approach was chosen to balance expert insight with unbiased evaluation, ensuring that the reliability, accuracy, and comprehensibility ratings reflected a broader clinical perspective.

Each panel member reviewed the responses independently and evaluated them along three Likert-scale dimensions including reliability (1–6 points), for consistency, trustworthiness, and apparent evidence-based underpinnings of the response; accuracy (1–3 points) for measuring alignment with up-to-date medical standards, guidelines, and pharmacological principles related to opioid therapy; and comprehensibility (1–3 points) which reflects the clarity, readability, and accessibility of the information for a general patient audience. Consistent with prior research, responses were deemed “acceptable” if they met or exceeded the following cutoff criteria: reliability ≥ 4, accuracy ≥ 2, comprehensibility ≥ 3 [8].

To capture diverse perspectives and ensure that patient-centric communication needs were met, non-healthcare participants specifically rated the readability and overall accessibility of the content [21]. An in-depth review process was also undertaken to appraise the coverage of clinical nuances, including references to individualized risk factors, tapering protocols, and monitoring strategies.

### 2.4. Word-Length Analysis

To evaluate the potential for “information overload”, the word count for each AI-generated answer was measured using standard text processing tools. Responses were then grouped according to the procedural phase—pre-therapy, during therapy, and post-therapy—and average word lengths were compared descriptively. The intent was to explore whether ChatGPT responses varied substantially in length across different types of patient inquiries and if this variability could influence patient comprehension or acceptance.

### 2.5. Statistical Analysis

All quantitative data were summarized as mean ± standard deviation (SD). Descriptive statistics were generated to identify patterns in ChatGPT’s performance across the 13 questions and to examine any notable differences in word length or evaluator ratings by domain. Inter-rater agreement was initially explored through qualitative comparison of participant feedback. More advanced statistical modeling, such as repeated-measures ANOVA or intraclass correlation, was considered to detect outliers and to assess the consistency of ratings across different evaluators.

Since no direct patient data were collected, this study was deemed exempt from the Institutional Review Board (IRB) review. All analyses were performed using IBM SPSS Statistics (version 26), and findings were interpreted in the context of existing literature on AI-driven patient education. The resulting insights aim to inform best practices for refining ChatGPT’s capabilities and guiding the integration of AI tools into clinical workflows for chronic pain management.

## 3. Results

The panel selected thirteen frequently asked questions concerning opioid long-term therapy. To organize the evaluation, the questions were stratified into three time-based domains: Pre-therapy: Questions 1–2; During therapy: Questions 3–8; Post-therapy: Questions 9–13 (Table 1).

According to the Copilot assessment for comprehensibility, the questions were judged comprehensible and well structured. Although the LLM suggested splitting question 9 into two separate questions for clarity, the panel decided to maintain the original version to avoid redundancy (Table 2).

From the ChatGPT output assessment, an amount of 36 data points per question were obtained. Across all evaluated responses, the overall mean reliability was 5.2 ± 0.6 (range: 4.7–5.6). Notably, **Question 3** (“*Is long-term opioid therapy addictive?*”) achieved the highest reliability score (5.6 ± 0.5), suggesting strong consensus on ChatGPT’s ability to address more straightforward patient concerns. In contrast, **Question 7** (“*What are the signs of opioid dependency?*”) received a lower reliability score (4.7 ± 0.8), indicating that the AI’s treatment of dependency indicators varied among evaluators (Table 3).

With respect to accuracy, the average rating was 2.7 ± 0.3 (range: 2.5–2.9). Responses to Question 3 again scored highly (2.9 ± 0.1), as did Question 9 (“*How long does long-term opioid therapy last, and what happens when it needs to be discontinued?*”), reflecting relatively robust alignment with current guidelines on addiction risk and treatment duration. By contrast, Question 6 (on tolerance), Question 8 (on risk-reduction measures), and Question 13 (on complications) displayed slightly lower accuracy (2.5 ± 0.4). This suggests that ChatGPT’s explanations for more technical or nuanced opioid topics may benefit from additional domain-specific refinement.

Comprehensibility was rated 2.8 ± 0.2 on average (range: 2.5–3.0), indicating that ChatGPT generally provided clear and approachable language for patients. Several responses—including Question 1 (“*What are opioids?*”) and Question 4 (“*What are the risks and benefits of long-term opioid therapy?*”)—achieved a perfect comprehensibility score of 3.0 ± 0.0. However, more complex questions, especially those related to opioid tolerance (Question 6), signs of dependency (Question 7), and managing complications (Question 13), tended to score lower in clarity (2.5–2.6), underscoring the need for simpler language or a more structured explanation of technical concepts.

**Figure 1:**
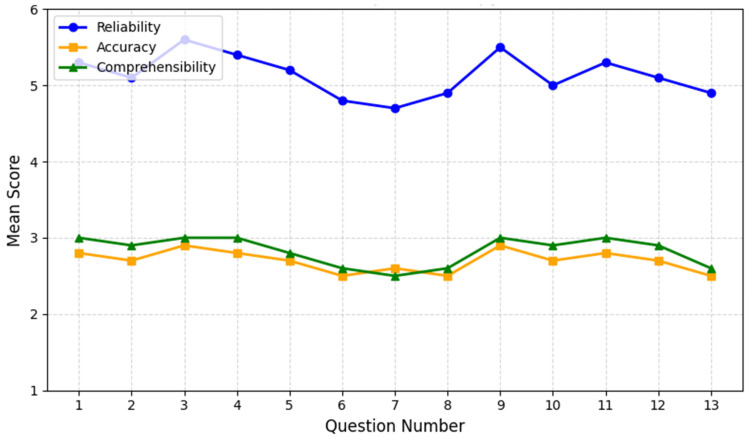
ChatGPT performance on opioid therapy questions. The chart depicts the mean scores for **reliability** (blue line), **accuracy** (orange line), and **comprehensibility** (green line) across 13 patient questions on long-term opioid therapy. The *X*-axis enumerates the questions from 1 to 13, while the *Y*-axis represents the average rating within each category. Overall, **reliability** remains above 4.7 for most questions, peaking for **Question 3** (“*Is long-term opioid therapy addictive?*”) at 5.6 ± 0.5. In contrast, **Question 7** (“*What are the signs of opioid dependency?*”) shows a lower reliability score (4.7 ± 0.8), reflecting variations in ChatGPT’s treatment of dependency indicators.

Most ChatGPT answers exceeded this study’s predefined acceptability thresholds of reliability ≥ 4, accuracy ≥ 2, and comprehensibility ≥ 3, highlighting the model’s promise as a supplemental patient education tool in chronic pain management (Figure 1).

Accuracy scores generally hover between 2.5 and 2.9, with the highest scores again observed for Question 3 and for Question 9 (“*How long does long-term opioid therapy last, and what happens when it needs to be discontinued?*”). More complex topics, such as tolerance (Question 6) and complications (Question 13), earned slightly lower accuracy scores, suggesting room for domain-specific refinement.

Comprehensibility averages around 2.8 ± 0.2, with perfect 3.0 ± 0.0 marks on simpler or more procedural questions (e.g., Question 1: “*What are opioids?*” and Question 4: “*What are the risks and benefits of long-term opioid therapy?*”). However, more nuanced issues—particularly related to tolerance (Question 6), dependency (Question 7), and complications (Question 13)—scored closer to 2.5–2.6, highlighting the need for clearer explanations of intricate medical concepts. Overall, the results indicate ChatGPT’s strong performance on fundamental questions and the necessity for further refinement when addressing specialized or technically challenging topics.

### Word-Length Analysis

To investigate the relationship between response length and patient comprehension, each AI-generated answer was analyzed for word count using standard text processing methods. As shown in Table 3, introductory questions—such as Q1 (“*What are opioids?*”) and Q2—tended to yield shorter responses (around 140–150 words) while maintaining high comprehensibility scores. Conversely, more complex or detailed topics—particularly Q7 (signs of opioid dependency) and Q13 (managing complications)—displayed increased word counts (exceeding 180 words) without a corresponding improvement in comprehensibility. These findings suggest that adding more information does not necessarily enhance clarity. Moving forward, carefully tailoring the amount of detail to balance thoroughness with patient-friendly language may be crucial, especially for questions that demand nuanced discussions of opioid-related risks.

## 4. Discussion

The results of this study reinforce generative AI promise as an adjunctive tool for patient education in long-term opioid therapy. The model consistently demonstrated high reliability (5.2 ± 0.6) and good comprehensibility (2.8 ± 0.2), suggesting that even complex medical concepts can be translated into user-friendly language that patients can readily understand. Similar trends have been noted in other fields, such as procedural pain management, where AI chatbots significantly enhanced patient comprehension by simplifying intricate medical concepts [8].

At the same time, limitations emerged in ChatGPT’s responses to specialized or nuanced questions, particularly those involving opioid tolerance (Question 6), dependency (Questions 7, 13), and risk-reduction strategies. These topics require up-to-date, domain-specific knowledge that may not be captured by a generalized language model [20]. Consistent with previous studies, LLMs often rely on publicly accessible data, potentially lacking detailed or cutting-edge medical information unless specifically trained on curated datasets [20,22]. Consequently, the knowledge cutoff (April 2023) of the ChatGPT-4.0 version used here may further restrict its relevance for evolving fields like opioid therapy, where new tapering schedules and non-opioid adjunct treatments continue to emerge [23,24]. This shortfall underscores the importance of regular dataset updates and tailored AI training for specialized healthcare applications [9,20]. Other high-stakes clinical domains similarly benefit from AI-driven solutions only when supplemented with comprehensive training and real-time updates. For example, studies in cardiogenic shock and pulmonary hypertension demonstrate that advanced AI analytics can enhance both diagnostic accuracy and therapeutic planning. Therefore, the underlying models are continually refined with the latest clinical data [25].

Cultural and linguistic adaptation likewise remains underexplored. Addressing diverse patient populations—where language nuances, health literacy levels, and cultural beliefs vary—demands that AI-based education tools incorporate culturally competent frameworks. In parallel, ethical and legal considerations become paramount when deploying AI in healthcare [9,10,26]. This is especially relevant in opioid therapy, where misinformation could lead to unsafe medication practices or exacerbation of misuse and addiction [27,28]. Although disclaimers can clarify that AI outputs do not replace professional medical advice, robust oversight mechanisms—including regulatory compliance (e.g., HIPAA) and potential FDA oversight of clinical decision support tools—are essential to ensure patient safety and provider accountability [29].

Despite these constraints, the advantages of AI in patient care and education remain significant, and other research has demonstrated that LLMs can effectively produce a clinical recommendation for complex pain issues such as low back pain [30]. These technologies could alleviate clinicians’ time demands for routine education, providing around-the-clock accessibility. Consequently, generative AI chatbots like ChatGPT can enable patients to become more informed participants in their own care [31,32,33]. This enhanced engagement can produce broad implications for improving adherence to treatment plans and overall satisfaction with care [34]. Importantly, some of the challenges that have been identified in opioid therapy—such as rapidly evolving prescribing guidelines—are also seen in other specialized contexts, reinforcing the broader need for ongoing multidisciplinary collaboration among healthcare professionals, AI developers, and regulatory entities to maintain high-quality, equitable, and up-to-date AI-driven tools [35].

Looking ahead, future research might explore ways to integrate ChatGPT with telehealth platforms or interactive patient portals, enabling real-time access to personalized education materials and further reducing barriers to care. Additionally, the development of domain-specific modules could enhance ChatGPT’s performance in addressing topics that require higher clinical nuance, such as the interplay between psychological support and pharmacological management in opioid use, across different populations suffering from cancer and non-cancer-related pain. Ultimately, the successful adoption of AI in chronic pain management will rely not only on technological improvements but also on robust frameworks for patient safety, data privacy, and ethical deployment, ensuring that AI-based education aligns seamlessly with the broader goals of personalized and compassionate care.

## 5. Limitations

Several limitations should be considered when interpreting these findings. First, the sample size (12 evaluators) may not capture the full diversity of perspectives, and a larger or more heterogeneous panel could yield different conclusions. Second, this study focused exclusively on ChatGPT-4.0, thereby omitting comparisons with alternative AI platforms [20]. Because each AI model leverages different data sources and training architectures, results from a single platform may not be fully generalizable. Nevertheless, we employed a second LLM (Copilot) to assess comprehensibility. Therefore, a cross-model evaluation was performed. Third, ChatGPT-4.0’s knowledge cutoff potentially restricts its ability to address newer developments, including updated opioid tapering protocols, evolving best practices, and novel adjunct therapies. As AI models continue to evolve, future work should employ the latest versions or verify the specific training data cutoff to ensure access to the most current medical knowledge.

Additionally, patient perspectives were not directly assessed, which limits the study’s insights into real-world usability and satisfaction. While the inclusion of non-healthcare participants helped approximate a lay audience, collecting feedback from actual patients would provide a more robust understanding of how AI-generated information translates into clinical decision-making and adherence. Furthermore, following this research framework, the needs of different groups of chronic pain patients might be addressed. Finally, it is important to recognize that the reported performance metrics reflect a snapshot of ChatGPT-4.0’s capabilities at the time of evaluation. Subsequent model updates or the release of newer AI solutions may shift reliability, accuracy, and comprehensibility outcomes, underscoring the need for ongoing validation and longitudinal studies that account for improvements in AI training data, architecture, and real-time responsiveness.

## 6. Perspectives

Integrating AI-driven chatbots like ChatGPT into routine clinical workflows for long-term opioid therapy holds substantial promise. Given their capabilities to deliver consistent, on-demand patient education, these technologies can help reduce clinicians’ administrative burden, streamline information dissemination, and improve patient engagement [30,31]. To unlock these benefits, stakeholders must ensure continuous updates of AI training data to reflect evolving prescribing guidelines and novel pain management strategies. Technological refinements are also required. For example, for pain management, generative AI methods can be combined with other AI-based approaches such as those pertinent to automatic pain assessment for building multidimensional models [36,37], for improving telehealth-centered care pathways [38], multimodal signal monitoring [39,40,41], and achieve other objectives [42].

Equally important is the adaptation of AI outputs to meet diverse linguistic, cultural, and health literacy needs, promoting accessibility for all patient populations [43]. On the other hand, close collaboration among AI developers, healthcare providers, and regulatory authorities will be essential to establish clear guidelines, maintain quality standards, and safeguard patient well-being [44]. With refinements, AI-driven tools may become invaluable partners in delivering personalized, evidence-based pain management, ultimately enhancing patient outcomes and satisfaction, as well as administrative efficiency [45].

## 7. Conclusions

This study demonstrates that a generative AI model can function as a valuable supplementary resource for patients undergoing long-term opioid therapy. This technology exhibits strong potential for enhancing patient education and encouraging proactive involvement in treatment decisions. Nonetheless, the model’s shortcomings in specialized opioid-related topics—such as complex tapering protocols or comorbidities—highlight the ongoing need for targeted refinements, frequent data updates, and oversight by clinicians well versed in pain management.

The effective integration of AI-driven education into clinical workflows will require close collaboration among healthcare professionals, AI developers, regulatory authorities, and patients. Key priorities include ensuring cultural and linguistic adaptability, maintaining ethical and legal safeguards, and implementing robust validation for AI-generated content. By operating as an adjunct—rather than a substitute—for physician input, AI-based tools can empower patients, bolster adherence, and support more personalized, evidence-based care in chronic pain management. Finally, continuous model training and domain-specific enhancements will be essential to keep pace with evolving clinical guidelines. This is a crucial step for improving long-term outcomes and patient satisfaction.

## Figures and Tables

**Table 1:** Common Patient Questions About Opioid Long-Term Therapy, Categorized by Procedural Domain.

Domain	Questions
Pre-Therapy	1. What are opioids? 2. How do opioids work in pain management?
During Therapy	3. Is long-term opioid therapy addictive? 4. What are the risks and benefits of long-term opioid therapy? 5. How can I manage the side effects of opioids? 6. How does opioid tolerance develop? 7. What are the signs of opioid dependency? 8. How can I reduce the risk of dependency during long-term opioid therapy?
Post-Therapy	9. How long does long-term opioid therapy last, and what happens when it needs to be discontinued? 10. Can I stop opioid therapy if necessary? 11. What activities should I avoid during long-term opioid therapy? 12. How soon can I return to normal activities after starting opioid therapy? 13. What should I do if I experience discomfort or complications with my long-term opioid therapy?

**Table 2:** Microsoft Copilot evaluation for comprehensibility.

Domain	Questions	Comprehensibility	Improvement
Pre-Therapy	1. What are opioids?	High	None needed.
Pre-Therapy	2. How do opioids work in pain management?	High	None needed.
During Therapy	3. Is long-term opioid therapy addictive?	High	None needed.
During Therapy	4. What are the risks and benefits of long-term opioid therapy?	High	None needed.
During Therapy	5. How can I manage the side effects of opioids?	High	None needed.
During Therapy	6. How does opioid tolerance develop?	High	None needed.
During Therapy	7. What are the signs of opioid dependency?	High	None needed.
During Therapy	8. How can I reduce the risk of dependency during long-term opioid therapy?	High	None needed.
Post-Therapy	9. How long does long-term opioid therapy last, and what happens when it needs to be discontinued?	Medium	Consider splitting into two questions: “How long does long-term opioid therapy typically last?” and “What happens when long-term opioid therapy needs to be discontinued?”
Post-Therapy	10. Can I stop opioid therapy if necessary?	High	None needed.
Post-Therapy	11. What activities should I avoid during long-term opioid therapy?	High	None needed.
Post-Therapy	12. How soon can I return to normal activities after starting opioid therapy?	High	None needed.
Post-Therapy	13. What should I do if I experience discomfort or complications with my long-term opioid therapy?	High	None needed.

**Table 3:** Reliability, Accuracy, Comprehensibility, and Word Count of ChatGPT Responses.

Q No.	Reliability (1–6) Mean ± SD	Accuracy (1–3) Mean ± SD	Comprehensibility (1–3) Mean ± SD	Word Count (Mean ± SD)
1	5.3 ± 0.5	2.8 ± 0.2	3.0 ± 0.0	145 ± 20
2	5.1 ± 0.6	2.7 ± 0.3	2.9 ± 0.1	140 ± 18
3	5.6 ± 0.5	2.9 ± 0.1	3.0 ± 0.0	155 ± 22
4	5.4 ± 0.6	2.8 ± 0.2	3.0 ± 0.0	160 ± 25
5	5.2 ± 0.7	2.7 ± 0.3	2.8 ± 0.2	170 ± 28
6	4.8 ± 0.9	2.5 ± 0.4	2.6 ± 0.3	175 ± 20
7	4.7 ± 0.8	2.6 ± 0.3	2.5 ± 0.4	180 ± 22
8	4.9 ± 0.9	2.5 ± 0.4	2.6 ± 0.3	185 ± 24
9	5.5 ± 0.5	2.9 ± 0.1	3.0 ± 0.0	160 ± 18
10	5.0 ± 0.7	2.7 ± 0.3	2.9 ± 0.1	170 ± 27
11	5.3 ± 0.6	2.8 ± 0.2	3.0 ± 0.0	165 ± 25
12	5.1 ± 0.7	2.7 ± 0.3	2.9 ± 0.1	175 ± 26
13	4.9 ± 0.8	2.5 ± 0.4	2.6 ± 0.3	190 ± 30

**Note:** Reliability ranges from 1 (lowest) to 6 (highest); accuracy ranges from 1 (lowest) to 3 (highest); comprehensibility ranges from 1 (lowest) to 3 (highest); word count represents the average number of words in ChatGPT’s response to each question, with its corresponding standard deviation.

## Data Availability

The raw data supporting the conclusions of this article will be made available by the authors on request.
